# Successful Laparoscopic Management of Chronic Diaphragmatic Hernia Post Trauma Masquerading As Intestinal Obstruction: A Case Series

**DOI:** 10.7759/cureus.78453

**Published:** 2025-02-03

**Authors:** Aditya Sharma, Mumtaz A Ansari, Vivek Srivastava

**Affiliations:** 1 General Surgery, Institute of Medical Sciences, Banaras Hindu University, Varanasi, IND

**Keywords:** blunt trauma abdomen, delayed presentation, laparoscopic hernia repair, subacute intestinal obstruction, traumatic diaphragmatic hernia

## Abstract

A rare and potentially fatal consequence of trauma is diaphragmatic rupture. Since severe concomitant injuries to other organs might obscure the signs and symptoms of acute diaphragmatic trauma, a high index of suspicion is necessary for clinical diagnosis, particularly in the absence of pathognomonic radiographic findings. Many clinical signs may appear years later in chronic situations. Traumatic diaphragmatic hernias are managed well with laparoscopic methods.

We report a case series of patients with an atypical presentation of subacute intestinal obstruction (SAIO) who, after further workup, were diagnosed with chronic traumatic diaphragmatic hernias and were successfully managed with laparoscopic hernia repair in our surgical unit.

## Introduction

The diagnosis and radiography of traumatic diaphragmatic rupture are still difficult, rare, and sometimes fatal. Many times, diagnosis is delayed, which increases the risk of disastrous outcomes [1,2]. The diagnosis and treatment of traumatic diaphragmatic hernia may benefit from laparoscopic intervention. A dangerous consequence of both blunt and penetrating abdominal injuries is diaphragmatic rupture. Diagnostic delays in acute cases can cause serious respiratory and cardiovascular impairment [3].

After trauma, patients may not show any symptoms for years until complications like SAIO arise, and diagnosis is typically delayed. Diaphragmatic injuries should be treated as soon as a diagnosis is made in order to lower the possibility of complications [4]. The conventional methods of treatment are laparotomy or thoracotomy, with the selection being based on the surgeon's competence level [3,4]. With the development of laparoscopy, this clinical scenario has changed. Nonetheless, there are few reports of laparoscopic or laparoscopic-assisted traumatic diaphragmatic hernia repair, and those that do exist are mostly restricted to congenital or chronic post-traumatic hernias [5].

## Case presentation

Case 1

A 22-year-old male presented to the emergency surgery department with the chief complaint of not being able to pass flatus and stools for the past two days. It was also associated with multiple episodes of vomiting and breathlessness. There was a history of blunt trauma to the abdomen following a road traffic accident a year ago. There is no history of any surgical history in the past. On examination, the abdomen was distended, with no local rise in temperature. Mild tenderness was present on palpation. On auscultation, sluggish bowel sounds were heard. 

On CT of the thorax, along with a defect in the left side (posterior aspect) of the diaphragm through which the abdominal contents were pushed inside the thorax and sequelae, leading to such a presentation. The patient was scheduled for elective laparoscopic repair of the hernia with mesh hernioplasty. Intraoperatively, mild adhesions were noted, the abdominal contents were the ileum along with part of the omentum, and part of the transverse colon was stuck inside the defect of 6x4 cm. The contents were viable and were reduced gently, and the defect was closed with the PDS 1-0 barbed suture by the intracorporeal suturing technique. Thereafter, the placement of a composite mesh was done. The patient did well during his postoperative period and was discharged on postoperative day three following an uneventful hospital stay. 

Case 2

A 58-year-old lady with distension in her abdomen and difficulty passing stool and flatus for the previous two days. It was also linked to several vomiting episodes. Three years prior, there had been a fall and the resulting acute abdominal damage. No prior history of surgical intervention is present. The abdomen appeared enlarged upon examination, though there was no localized increase in temperature. Auscultation revealed slow bowel sounds. CT thorax showed a diaphragmatic hernia on the left side.

The patient was scheduled for mesh hernioplasty, an elective laparoscopic procedure to correct the hernia. Mild adhesions were observed during surgery; the abdominal contents included the sigmoid colon, distal ileal segments, and a portion of the omentum lodged inside the 8x4 cm defect. After carefully reducing the contents which were viable, intracorporeal suturing was used to seal the defect using the PDS 1-0 barbed suture. The installation of a composite mesh was then completed. After an uneventful hospital stay, the patient did well during her postoperative period and was discharged on the fourth postoperative day.

Case 3

The primary complaint of a 48-year-old gentleman who presented at the emergency surgery department was that he had been unable to pass feces and flatus for the previous two days. Multiple episodes of nausea and vomiting were also linked to it. Following a car accident two years ago, there was a history of blunt trauma to the abdomen. No prior history of surgical intervention was present. Upon examination, there was no localized increase in temperature, though the abdomen was enlarged. Auscultation revealed slow bowel movements.

A left-sided traumatic diaphragmatic hernia was discovered by CT thorax imaging. The patient was scheduled for mesh hernioplasty, an elective laparoscopic procedure to correct the hernia. Mild adhesions were observed intraoperatively; the ileum and a portion of the omentum were found in the abdominal contents, and a portion of the transverse colon was lodged inside the 8x4 cm defect. After carefully reducing the contents which were viable, intracorporeal suturing was used to seal the defect using the PDS 1-0 barbed suture. The installation of a composite mesh was then completed. After an uneventful hospital stay, the patient did well during his postoperative period and was released on the third postoperative day.

**Figure 1:**
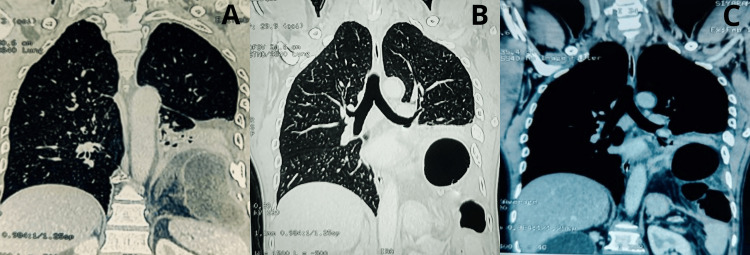
CT of the thorax, along with a defect in the left side (posterior aspect) of the diaphragm along with the abdominal contents A-C: Left-side traumatic diaphragmatic hernia in Cases 1, 2, and 3, respectively

CT images of all three patients have been shown in Figure 1.

## Discussion

An abdominal content or organ herniating into the thoracic cavity due to weakening or a breach in the diaphragm is known as a diaphragmatic hernia [6]. Rarely, blunt (2.9%) or penetrating (3.4%) trauma to the thoracic or abdominal cavity results in traumatic diaphragmatic injuries. The actual frequency of this injury, however, is probably underreported. Diagnostic imaging frequently misses diaphragmatic injuries when there are no acute presentations. Making the diagnosis might be challenging because imaging may initially fail to identify the injury, and the physical examination may be unremarkable [3,4,6].

Due to the left diaphragm's inherent weakness compared to the right diaphragm's protection by the liver, the majority of severe diaphragmatic injuries (80-90%) affect the left diaphragm [7]. Patients may present asymptomatic, develop an acute presentation such as dyspnea, epigastric discomfort, shortness of breath, or vomiting, or develop an advanced clinical presentation such as intestinal obstruction, strangulation, or perforation once adhesions have developed. The presence of pulmonary disease, the size of the diaphragmatic defect, and herniated organs are all significantly correlated with the symptoms and indicators for surgical outcomes [4,5].

The majority of casualties will have a chest radiograph obtained as part of the first trauma assessment. The chest radiograph is frequently the first indicator of the existence of diaphragmatic injury and is considered a crucial supplementary tool in the Advanced Trauma Life Support guidelines for the initial assessment of the trauma patient. A chest radiograph, however, would not always be helpful because concomitant lung contusions, hemothorax, pneumothorax, pleural effusions, atelectasis, emphysema, and non-specific elevation of the diaphragm can obscure symptoms [6]. A CT scan of the chest is now a necessary diagnostic tool for trauma patients who are hemodynamically stable. Repairing traumatic diaphragmatic hernias requires adherence to two principles: total reduction of the herniated organs back into the abdomen and watertight closure of the diaphragm to prevent recurrence. In most circumstances, non-absorbable basic sutures are sufficient for repair; large, persistent defects are best served by using a mesh [3,4,6].

The synthetic or biologic meshes can be utilized to treat large diaphragmatic hernias in patients without creating tension and to prevent recurrence. Always keep in mind that while prostheses can help heal chronic diaphragmatic injuries, there is a significant risk of infection in the acute setting. Due to these factors, laparoscopic hernia repair techniques have grown in favor as a superior diagnostic and therapeutic option when diaphragmatic rupture is anticipated [5,7].

Traumatic rupture-related diaphragmatic hernia is an uncommon condition that can develop following acute abdominal trauma or stab wound injuries. We aimed to report successful management of diaphragmatic hernias secondary to traumatic injuries by using synthetic meshes as seen in the series of cases that presented with obstruction and had favorable outcomes after surgical management.

## Conclusions

Diaphragmatic injuries are uncommon and have a high morbidity and death rate, which is typically brought on by concomitant injuries and a delayed diagnosis. Diaphragmatic rupture cannot be diagnosed with specific signs and symptoms; a high index of suspicion is necessary. There are few reports of laparoscopic diaphragmatic rupture repairs. In patients who are hemodynamically stable, laparoscopy should be carried out as a diagnostic and therapeutic modality. For certain individuals with traumatic abdominal damage, it is a safe and successful way to repair the diaphragm.
